# E-cigarette and Vaping-Induced Lung Injury (EVALI) Long Taken for Miliary Tuberculosis: A Rare Cause of Interstitial Lung Disease

**DOI:** 10.7759/cureus.47948

**Published:** 2023-10-30

**Authors:** Chaynez Rachid, Oussama Fikri, Lamyae Amro

**Affiliations:** 1 Pulmonology Department, Hôpital Arrazi, CHU Mohammed VI, Laboratoire de Recherche Morpho Sciences, Faculté de médecine et de Pharmacie de Marrakech, Université Cadi Ayyad (Labo LRMS, FMPM, UCA), Marrakech, MAR

**Keywords:** micronodules, organized pneumonia, corticoids, e-cigarette associated lung injury, smoking-related diffuse interstitial lung disease

## Abstract

Electronic cigarettes, a recent and burgeoning product, are gaining traction among the general population. However, despite their growing popularity, there is a lack of comprehensive research on their potential health risks. A prominent concern is EVALI (electronic cigarette or vaping product use-associated lung injury), a newly recognized condition currently under intense investigation. Here, we report the case of a 24-year-old male with a history of chronic smoking e-cigarettes and vaping products heavily over the past year. He sought urgent care at the emergency room due to symptoms that had been present for seven days before seeking medical attention. These symptoms included a sudden onset of difficulty breathing at rest, an intermittent dry cough producing a small amount of greenish sputum, and occasional episodes of mild hemoptysis. Chest radiograph showed bilateral diffuse infiltrates including almost innumerable tiny interstitial nodules. Multiple lobes of the lungs were affected by consolidations and patches of ground-glass opacities in the chest high-resolution computed tomography (HRCT) image. Throughout the following week, the patient's health showed gradual improvement with the aid of supportive measures and corticosteroid treatment. As part of the recovery plan, the patient was released with a gradually reducing regimen of oral corticosteroids and was scheduled for regular outpatient monitoring. The progression of the recovery was notable through enhancements in clinical symptoms, biological markers, and radiological findings.

## Introduction

The popularity of e-cigarettes, also known as vaping, has surged in recent times. While these devices were initially presented as a less harmful substitute for conventional cigarettes, the recent revelation that vaping can lead to sudden lung issues has shattered the perception of their harmlessness. A substantial number of individuals, mainly teenagers and young adults, have experienced acute lung injury (ALI) due to vaping, a condition now termed e-cigarette or vaping-induced lung injury (EVALI). Here, we report a case of smoking-related diffuse interstitial lung disease with distinct and confusing radiological and clinical presentation in a young male patient. Through this work, we emphasize the rarity of this form, the difficulty of making an early diagnosis, and the importance of considering it early. Finally, we discuss the excellent response of this unusual form of smoking related diffuse interstitial lung disease to corticosteroid therapy and the importance of early therapy.

## Case presentation

**Figure 1:**
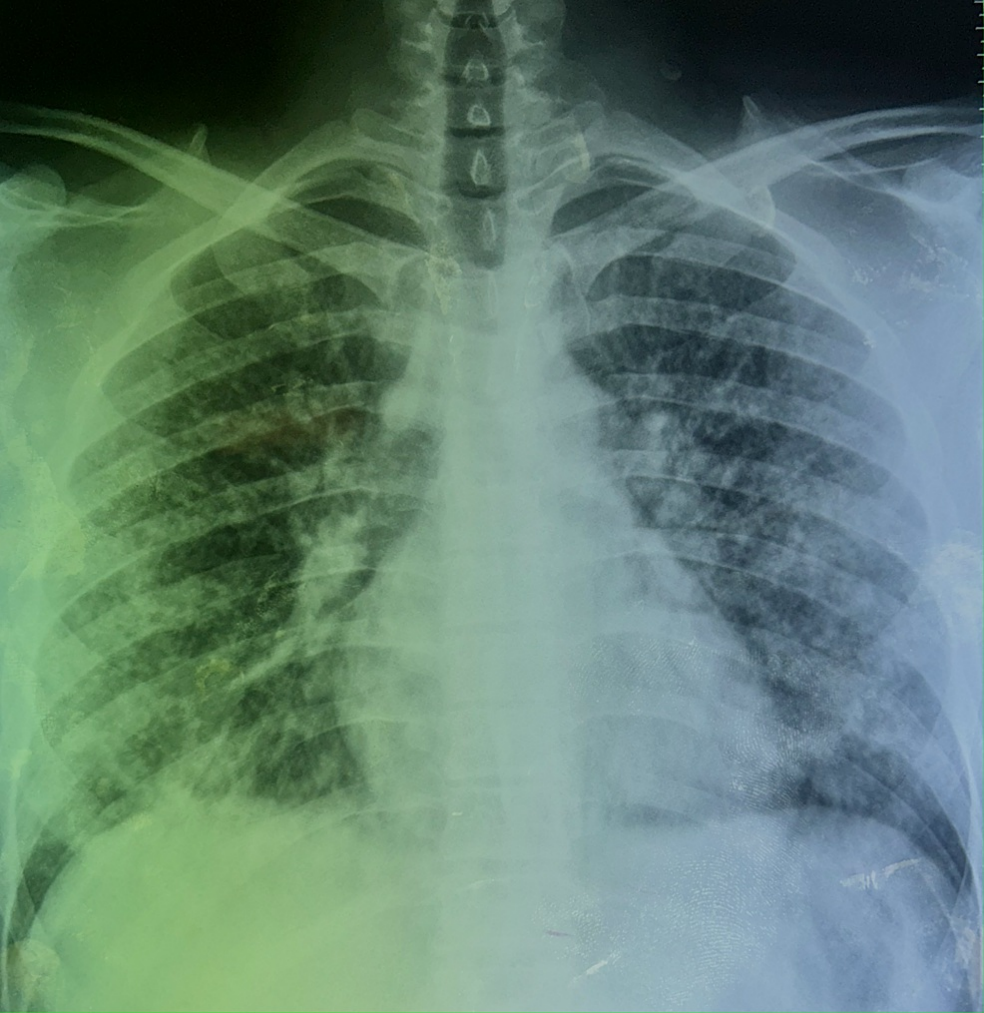
Posteroanterior chest radiograph demonstrates signs suggestive of miliary tuberculosis The image shows almost innumerable tiny interstitial nodules (e.g., 2-3 mm) distributed in both lungs some of which are confluent in places.

The patient is a 24-year-old male, waiter by profession, with no history of tuberculosis or no recent tuberculosis contagion, chronic smoker seven years unweaned, hashish user for five years weaned, occasional alcohol user, notion of protected sex. He denied any recent travel, sick contacts but reported using e-cigarettes and vaping products heavily over the past year. He consulted the emergency room for a symptomatology that appears to date back seven days prior to non-admission, characterized by acute onset of rest dyspnea, dry cough intermittently productive of sputum of small greenish abundance with episodes of hemoptysis of small abundance associated with diffuse bilateral chest pain. In the context of seven-kilogram weight loss and unquantified feverish sensations, all of this is involved. On examination, the patient appeared acutely ill and was in respiratory distress. Vital signs revealed a temperature of 38.5°C, heart rate of 105 beats per minute, respiratory rate of 30 breaths per minute, and oxygen saturation of 88% on room air and performans status at one. Lung auscultation revealed diffuse crackles and decreased breath sounds bilaterally. Chest radiograph showed bilateral diffuse infiltrates including almost innumerable tiny interstitial nodules distributed in both lungs some of which are confluent in places (Figure 1).

**Figure 2:**
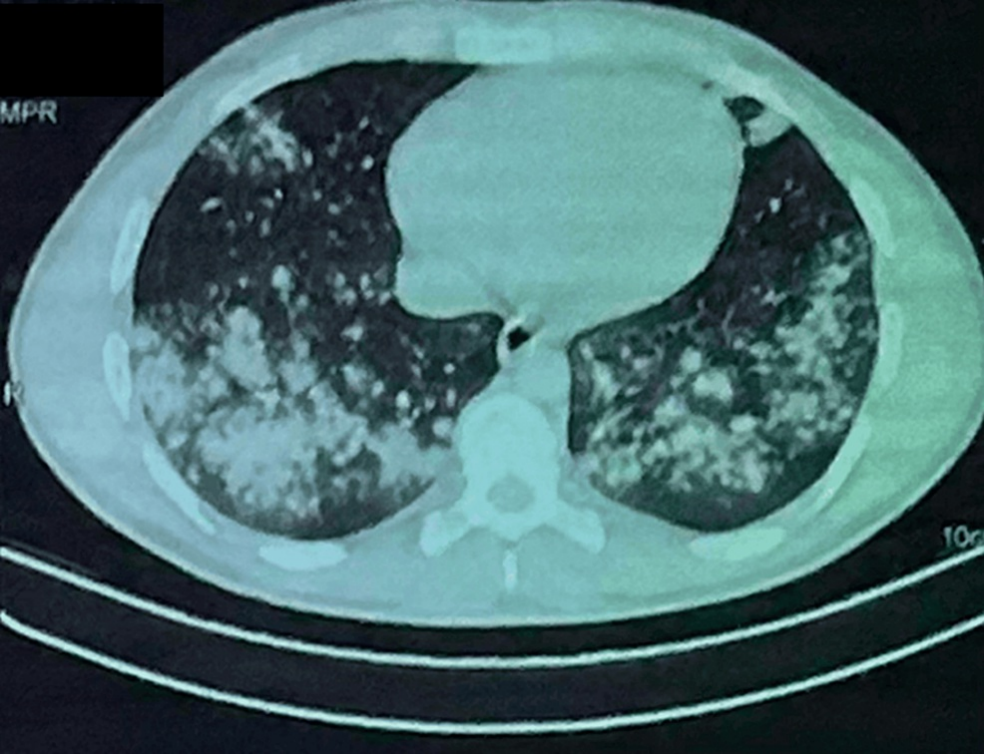
- Chest computed tomography scan in the parenchymal window The image is showing atypical patterns in e-cigarette or vaping-induced lung injury (EVALI) who presented with fevers and night sweats for 1 week. The CT can image shows spectrum of lung injury associated with centrilobular nodules (CNs) associated with a diffuse organizing pneumonia (OP) pattern of EVALI with diffuse ground-glass opacity (GGO) with subpleural and peribronchovascular (PBV) sparing.

The hemogram showed hyperleukocytosis of 25020/mm^3^, neutrophils of 19960/ mm^3^ and lymphocytes of 3060/mm^3^. Sputum microscopy and sputum Xpert gene were negative. Bronchoscopy appearance was unremarkable, desaturation on suction maneuvers. The search for an acid-alcohol- resistant bacillus in the bronchial aspirates was negative, quantiferon was doubtful, *Pneumocystis jirovecii* on induced sputum was negative. Pneumococcal antigenuria was positive. Testing for common respiratory viruses, including influenza and respiratory syncytial virus, was also negative. Arterial blood gas analysis indicated respiratory acidosis with hypoxemia. High-resolution computed tomography (HRCT) of the chest demonstrated ground-glass opacities and consolidations involving multiple lung lobes (Figure 2).

**Figure 3:**
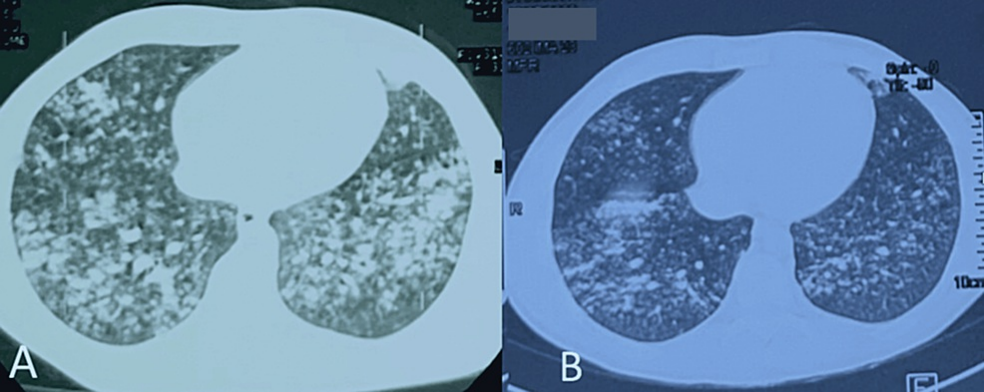
Thoracic CT scan showing spectrum of lung injury associated with centrilobular nodules (CNs) in e-cigarette or vaping product use-associated lung injury The CT scan image confirms multiple bilateral pulmonary micronodules of bronchogenic diffuse centrilobular nodularity, with discret regression, sparing the subpleural region and confluent in places (A) combined with the onset of radiological clean-up after one week of oral corticosteroid treatment (B).

Based on the clinical presentation, radiological findings, and exclusion of infectious etiologies, a diagnosis of e-cigarette and vaping-induced lung injury (EVALI) was suspected. The patient's history of heavy e-cigarette and vaping product use further supported this diagnosis. The patient was immediately started on supplemental oxygen, and broad-spectrum antibiotics to cover potential bacterial infections. Supportive measures, including intravenous fluids and administation of glucorticosteroids, cessation of vaping was initiated. The patient was put on long-term oxygen therapy 4 L/ min, corticotherapy 20 mg: three times a day, and antibiotic therapy (amoxicillin + clavulanic acid: 3 g/day. After 14 days of treatment, clinical, biological improvement and radiographic changes were noted (Figure 3). There was an improvement in respiratory rate and saturation from 88% to 93% in room air, white blood cells from 25020/mm^3^ to 16000/mm^3^ and CRP from 305.58 to 18 and radiological cleaning with persistence of bronchogenic micronodules with subpleural border. HRCT confirms multiple bilateral pulmonary micronodules (A) with radiological improvements after one week of oral corticosteroid treatment (B) (Figure 3).

**Figure 4:**
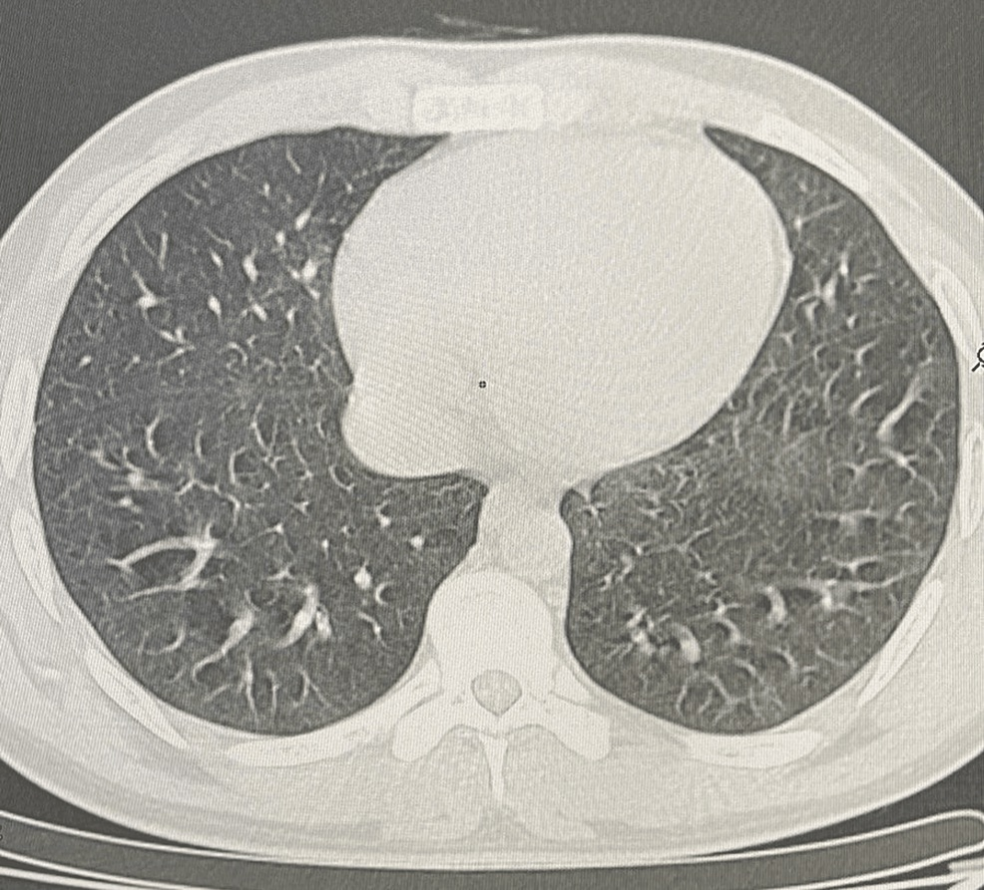
Scannographic clean up Thoracic CT scan showing absence of progressive pleuropulmonary lesions

Similar imaging and pathologic findings have been described in patients with smoke synthetic cannabinoids. Over the next week, the patient's condition improved gradually with supportive care and corticosteroid therapy. He was discharged with a tapering course of oral corticosteroids and close outpatient follow-up. The evolution was marked by clinical, biological and radiological improvement after two months of corticosteroid therapy (Figure 4).

## Discussion

This case highlights the severity and complexity of EVALI, a condition associated with the use of e-cigarettes and vaping products. EVALI can present a range of symptoms, from mild respiratory distress to severe acute respiratory failure. Early recognition, cessation of vaping, and appropriate management are essential for positive outcomes. Physicians and healthcare providers should maintain a high index of suspicion for EVALI, especially in young patients with a history of e-cigarette and vaping product use, and promptly initiate diagnostic and therapeutic interventions.

Adolescents and young adults bear a disproportionate burden of EVALI, constituting 19% of the US population over 12 years old, yet accounting for slightly over half of the reported EVALI cases. Moreover, there are notable distinctions in substance use patterns and clinical traits between adolescents, young adults, and older adults affected by EVALI. The majority of adolescents with EVALI disclosed using products containing tetrahydrocannabinol (THC), often sourced informally from family, friends, or in-person and online dealers, a trend more pronounced than in their older counterparts. Additionally, adolescents with EVALI exhibited a higher prevalence of prior attention deficit hyperactivity disorder (ADHD) diagnoses compared to young adults and adults with EVALI. Asthma history was also more frequently reported among adolescents with EVALI than adults with the condition. Notably, gastrointestinal and constitutional symptoms were more prevalent during the initial clinical presentation for adolescents, potentially complicating diagnosis and treatment timelines for EVALI. These insights are crucial for shaping the public health and clinical approach to addressing EVALI within the teenager’s population. The majority of adolescents affected by EVALI (81.7%) acknowledged using e-cigarette or vaping products containing THC, and an overwhelming proportion (96.5%) reported procuring these THC-containing items through informal channels such as family, friends, or in-person and online sellers. This trend of informal acquisition was notably more prevalent among adolescents with EVALI compared to their young adult and adult counterparts. Additionally, a significant proportion of adolescents (57.9%) reported using THC-containing products on a daily basis [1].

This pattern aligns with earlier nationwide findings and reinforces the notion that THC-containing e-cigarette or vaping products are strongly linked to the majority of EVALI cases, playing a pivotal role in the 2019 EVALI outbreak. This is substantiated by the discovery of vitamin E acetate, a compound linked to EVALI, in informally sourced THC-containing e-cigarette or vaping products obtained from various states across the country. However, investigations are ongoing to ascertain other potential culprits, including chemicals present in both THC and non-THC products, as the definitive cause of EVALI is still undetermined [2]. Multiple factors could be contributing to the onset of EVALI. The frequent presence of specific comorbidities among adolescents grappling with EVALI holds valuable implications for clinical assessment and intervention strategies. In this study, we observed a higher prevalence of ADHD among adolescents with EVALI compared to the general adolescent population (18.1% vs. 10.5%), highlighting a notable trend. Adolescents with EVALI reported a history of ADHD at a rate 2 to 4 times higher than young adults or adults afflicted by EVALI (18.1% vs. 7.9% and 4.9%, respectively). Additionally, ADHD has been linked to engaging in risky behaviors, such as e-cigarette use, further emphasizing its relevance in this context. Approximately half of the adolescents with EVALI disclosed a history of concurrent behavioral health conditions, which aligns with the broader patient population affected by EVALI. Considering and addressing these conditions is crucial to optimize clinical care and make appropriate referrals to behavioral health services, potentially mitigating the risk of subsequent substance use disorder [3,4].

Although the exact cause is uncertain, it's plausible that the distinct usage patterns of e-cigarette products by adolescents may contribute. Compared to adults, adolescents may lack experience with e-cigarettes and inhale less deeply, potentially leading to increased ingestion of e-cigarette aerosol and heightened exposure of aerosol components to the gastrointestinal system. Further studies are required to investigate this hypothesis. The presence of general gastrointestinal and constitutional symptoms could complicate the timely identification of EVALI, as it primarily relies on exclusion-based diagnosis [5]. A crucial aspect in ensuring prompt EVALI diagnosis involves obtaining a comprehensive, confidential substance use history and providing appropriate patient education, particularly regarding e-cigarette or vaping product usage. These results underscore the vital role of clinicians in educating and providing necessary services to adolescents. The educational efforts should focus on (1) highlighting the link between using e-cigarette or vaping products containing THC and EVALI, (2) emphasizing the presence of other potentially harmful chemicals, not limited to vitamin E acetate, even in products without THC, associated with EVALI, and (3) advising adolescents that using any e-cigarette or vaping product is unsafe [6,7]. Furthermore, our findings emphasize the urgent need for easily accessible tobacco cessation programs, substance use screening, and mental health services for all young individuals. Clinicians should be aware that adolescents frequently report vague gastrointestinal and constitutional symptoms, potentially expediting the prompt identification of EVALI [8].

## Conclusions

E-cigarette and vaping-induced lung injury (EVALI) is a serious pulmonary condition linked to the use of e-cigarettes and vaping products. This case report underscores the importance of early diagnosis, appropriate management, and public health efforts to educate individuals about the potential risks associated with vaping. Healthcare providers play a crucial role in recognizing EVALI, particularly in patients with a history of vaping and respiratory symptoms, in order to provide timely and effective interventions.
